# Impact of galcanezumab on total pain burden: findings from phase 3 randomized, double-blind, placebo-controlled studies in patients with episodic or chronic migraine (EVOLVE-1, EVOLVE-2, and REGAIN trials)

**DOI:** 10.1186/s10194-020-01190-7

**Published:** 2020-10-17

**Authors:** Jessica Ailani, J. Scott Andrews, Mallikarjuna Rettiganti, Robert A. Nicholson

**Affiliations:** 1 Georgetown University, 3800 Reservoir Rd NW, Washington, DC, 20007 USA; 2 Eli Lilly and Company, Indianapolis, IN USA

**Keywords:** Migraine, Chronic migraine, Burden of illness, Galcanezumab, Headache, Pain, Prevention, Clinical trial

## Abstract

**Background:**

Focus on the frequency of migraine pain may undervalue the total burden of migraine as pain duration and severity may present unique, additive burden. A composite measure of total pain burden (TPB; frequency, severity, and duration) may provide a more comprehensive characterization of pain burden and treatment response in patients with episodic migraine (EM) or chronic migraine (CM). The impact of galcanezumab versus placebo on TPB among patients with EM or CM was analyzed.

**Methods:**

Patients from randomized, double-blind, placebo-controlled episodic (two 6-month studies pooled) and chronic migraine (3-month) studies received once-monthly subcutaneous injection of galcanezumab 120 mg or placebo. A post hoc analysis of TPB for a given month was calculated as severity-weighted duration by multiplying duration (hours) and maximum pain severity (0 = none, 1 = mild, 2 = moderate, 3 = severe) of migraine for each day and summing these over the days in a month. Least square mean change from baseline in monthly TPB across Months 1–6 (EM, *N* = 444 galcanezumab, *N* = 894 placebo) and Months 1–3 (CM, *N* = 278 galcanezumab, *N* = 558 placebo) were compared using a mixed-model repeated measures model. Correlation of the Migraine Specific Quality of Life Questionnaire (MSQ) and Migraine Disability Assessment Scale (MIDAS) to TPB at baseline was assessed.

**Results:**

At baseline, the duration of migraine on a given migraine headache day accounted for the greatest unique proportion of variability (EM, 57.4% and CM, 61.1%) to TPB after adjusting for frequency of migraine headache days and maximum pain severity. The decrease from baseline in monthly TPB was greater with galcanezumab than placebo for patients with EM (68.6 versus 36.2) and CM (102.6 versus 44.4). The average percent reduction of TPB from baseline was significantly greater with galcanezumab compared with placebo in patients with EM (50.8% versus 17.2%) and CM (29.7% versus 11.0%). In patients with EM and CM, TPB correlated with MSQ total score (*r* = − 0.35 and *r* = − 0.37) and MIDAS (*r* = 0.34 and *r* = 0.32).

**Conclusions:**

Greater reduction in TPB was seen in patients with EM and CM treated with galcanezumab 120 mg once-monthly injection relative to placebo. Discussing TPB supports patient-centric conversations regarding treatment expectations when clinicians are evaluating options for migraine prevention.

**Trial registration:**

ClinicalTrials.gov: #NCT02614183 (I5Q-MC-CGAG; EVOLVE-1), #NCT02614196 (I5Q-MC-CGAH; EVOLVE-2), and #NCT02614261 (I5Q-MC-CGAI; REGAIN) – all 3 trials were registered on 23 November 2015.

## Background

It is well established that migraine can interfere with the occupational, educational, household, family, and social facets of daily life [1–10]. Consideration of the burden of migraine often focuses solely on the frequency of migraine pain; doing so may undervalue the total burden of migraine as pain duration and severity may present unique, additive burden [3, 6, 8, 9]. Total pain burden of migraine has been conceptualized as a composite measure involving frequency, duration, and severity. However, as novel therapies designed for the preventive treatment of migraine have become available, little research has focused on the additional burden of severity and duration. Evaluating this composite measure may be more aligned to the personal pain experience in migraine and could be useful in supporting patient-centric discussions regarding treatment expectations when clinicians are evaluating options for migraine prevention.

Galcanezumab is a humanized IgG4 monoclonal antibody that binds calcitonin gene-related peptide (CGRP) and prevents its biological activity without blocking the CGRP receptor and is indicated for the preventive treatment of migraine [11]. The efficacy of galcanezumab, as a preventive treatment for migraine, has been established across three randomized, double-blind, placebo-controlled, Phase 3 studies in patients with episodic migraine (EVOLVE-1 and EVOLVE-2) and chronic migraine (REGAIN). In those studies, galcanezumab-treated patients with episodic migraine experienced 4.3 to 4.7 fewer migraine headache days/month (versus 2.3 to 2.8 with placebo), and patients with chronic migraine had 4.8 fewer migraine headache days/month (versus 2.7 with placebo) [12–14].

The efficacy of galcanezumab for reduction of migraine frequency is established. However, it is unclear whether galcanezumab impacts total pain burden of migraine over time. Indication of this would provide greater clarity to providers regarding the overall potential clinical value of galcanezumab. In this post hoc study, total pain burden was compared between those taking galcanezumab 120 mg once-monthly injection (with an initial 240 mg loading dose) and placebo among patients with episodic or chronic migraine.

## Methods

### Study design

This post hoc analysis of Phase 3, randomized, double-blind, placebo-controlled studies in adult patients utilized data from two, 6-month episodic migraine studies pooled (EVOLVE-1 and EVOLVE-2) and one 3-month chronic migraine study (REGAIN) to analyze the impact of galcanezumab 120 mg once-monthly injection relative to placebo on total pain burden among patients with episodic migraine or chronic migraine [12–14]. Patients enrolled in the studies were 18 to 65 years of age and had a diagnosis of migraine (with or without aura) for at least 1 year prior to enrollment and onset prior to 50 years of age [12–15].

In all studies, patients entered a prospective baseline period of 30 to 40 days in which they completed an electronic patient-reported outcome diary to record the occurrence of headaches, headache duration, headache features, severity of headache, and use of headache medication. Eligible patients were randomized (1:1:2) to subcutaneous injections of galcanezumab 120 mg/month (following an initial 240 mg loading dose), galcanezumab 240 mg/month, or placebo. For the episodic migraine studies, patients were to have discontinued the use of medication or other treatments for the prevention of migraine for at least 30 days, and use of botulinum toxin A and B for at least 4 months, prior to the prospective baseline period. For the chronic migraine trial, patients were allowed to continue using topiramate or propranolol if they were on a stable dose in the 2 months prior to the prospective baseline period and remained on that dose throughout the baseline and double-blind periods. During all three studies, patients were permitted to continue acute migraine medications including triptans, ergots, nonsteroidal anti-inflammatory drugs, aspirin, and acetaminophen. Patients were permitted to continue acute migraine medications including triptans, ergots, nonsteroidal anti-inflammatory drugs, aspirin, and acetaminophen. Opioid- and barbiturate-containing medications were limited to 3 days/month, and only one corticosteroid injection was allowed during any period. Exclusion criteria of note for all three studies included, but was not limited to, prior exposure to any CGRP antibody or any therapeutic antibody 12 months prior to screening, using opioids or barbiturates more than twice per month, persistent daily headache, cluster headache, head or neck trauma within the past 6 months, possible posttraumatic headache, primary headache, or a medical or psychiatric illness that would preclude study participation [12–14]. The study protocols were reviewed and approved by the appropriate institutional review board for each of the study sites. The studies were conducted according to Good Clinical Practice and the Declaration of Helsinki guidelines. Patients provided written informed consent before undergoing study procedures. The studies are registered with ClinicalTrials.gov (NCT02614183, NCT02614196, and NCT02614261).

### Outcomes and statistical methods

The primary outcome of the EVOLVE-1, EVOLVE-2, and REGAIN studies was the overall mean change from baseline in the number of monthly migraine headache days during the double-blind treatment phase. This post hoc analysis evaluated the galcanezumab 120 mg and placebo treatment arms of the 3 studies. The objective of these analyses was to derive and compare between treatment groups, a “total pain burden” measure that incorporates: *frequency* of migraine headache days in a month, *duration* of migraine headache on a given day, and maximum *severity* of migraine headache on a given day. The total pain burden for a given month (severity-weighted duration) was calculated by multiplying duration (hours) of migraine headache and maximum pain severity (0 = none, 1 = mild, 2 = moderate, 3 = severe) for each migraine headache day and summing these over the days in a month. As an example, consider a patient who has 2 days of migraine headache in a month. The patient reports 2 h of migraine headache on Day 1, which is of mild severity (score = 1) and 3 h of migraine headache on Day 2, which is of moderate severity (score = 2). The total pain burden score for that month would be calculated as the sum of (2 h × 1) and (3 h × 2) which equals 8 severity-weighted hours of total pain burden. The change from baseline in monthly total pain burden measure over the double-blind period was analyzed for both episodic (6 months) and chronic migraine (3 months) studies using a mixed-model repeated measures (MMRM) model. The analysis of the episodic migraine studies included the following fixed effect variables: baseline, treatment, month, study indicator (EVOLVE-1 or EVOLVE-2), pooled region/country (nested within study), and the treatment by month, and baseline by month interaction effects. For the chronic migraine study, the fixed effect variables included: baseline, treatment, month, pooled country, baseline medication overuse (yes/no), concurrent prophylaxis use (yes/no), the interaction effects of treatment by month, and baseline by month. A marginal unstructured covariance structure was assumed to account for the correlation induced due to repeated measures on patients. The mean percentage change in total pain burden was analyzed using an MMRM model, as described earlier, for the change from baseline analyses. A similar MMRM modeling approach was also used to analyze change from baseline in each of the individual components of frequency of migraine headache days, duration per migraine headache day, and severity.

The Type II squared semi-partial correlation of each individual component within the total pain burden measure at baseline was obtained from the following regression model on the log transformed variables: log (total pain burden) = log (monthly migraine headache days), log (mean hours per migraine headache day), and log (severity of remaining migraine headache days). These provide the additional or unique proportion of variability explained by each component in its ability to predict total pain burden after adjusting for the other two components.

To explore the construct validity of the total pain burden measure, the Spearman’s rank correlation was used to determine the degree of correlation of the Migraine Specific Quality of Life Questionnaire (MSQ) and Migraine Disability Assessment Scale (MIDAS) to total pain burden at baseline. The MSQ is a self-administered instrument that evaluates the physical and emotional limitations of specific concern to patients with migraine [16]. The MIDAS quantifies days with headache-related disability across 5 areas over the last 3 months (90 days) [17].

#### Sensitivity analyses

The pain severity scores (0 = none, 1 = mild, 2 = moderate, 3 = severe) can be thought of as arbitrary. Therefore, a sensitivity analysis was performed by calculating (and comparing between groups) the total pain burden score using the square root of severity score in the calculation instead of severity (that is, using scores of 1 = mild, 1.414 = moderate, and 1.732 = severe) and using the square of the severity score in the calculation instead of severity (that is, using scores of 1 = mild, 4 = moderate, and 9 = severity). To further understand whether the changes observed in total pain burden can be fully explained by the change observed in frequency of migraine headache days, the original MMRM model analyzing change from baseline in total pain burden was repeated after including an additional time-varying covariate of the change from baseline in the number of monthly migraine headache days.

#### General considerations

Only patients in the intent-to-treat (ITT) population were considered for inclusion in this post-hoc analysis. Baseline demographics and descriptive summaries were provided for continuous variables using means and standard deviation and/or median and quartiles; for categorical variables, frequency and percentages were used. Treatment effects from MMRM models were expressed as least squares (LS) means and 95% confidence intervals (CI). No specific methods, such as multiple imputation, were employed to handle missing data. Furthermore, all repeated measures models included patients that had data at baseline and at least one time point during the double-blind phase. All statistical tests done were two-sided assuming a significance level of 5%. All statistical analyses were done using SAS^®^ Enterprise Guide version 7.1. All analyses done were post hoc and results should be considered exploratory.

## Results

### Patient disposition

Only patients in the ITT population from the placebo and the galcanezumab 120 mg arms of the pooled episodic EVOLVE-1 and EVOLVE-2 studies (total of 1338 patients; placebo *N* = 894, galcanezumab 120 mg *N* = 444) and the chronic migraine REGAIN study (total of 836 patients; placebo *N* = 558, galcanezumab 120 mg *N* = 278) were included in this post hoc analysis. The general baseline demographics and characteristics were balanced between treatment groups in the two episodic and one chronic migraine studies and have been previously well-described [12–14]. Relevant characteristics for the episodic and chronic migraine populations by treatment group are shown in Table 1. Among the three studies, patients were a mean age of 41 years, and the majority were female, White, and from North America. The mean number of migraine headache days/month among patients with episodic migraine in both treatment groups was 9.1 days and among patients with chronic migraine was 19.6 days for placebo and 19.4 days for galcanezumab. At baseline, both populations had a mean MIDAS total score indicative of severe disability and an MSQ total score indicative of migraine impacting quality of life at a level consistent with a patient presenting for migraine care. In the prospective baseline period (30 to 40 days), the mean total pain burden across both treatment groups was 124.3 severity-weighted hours for patients with episodic migraine and 320.4 severity-weighted hours for patients with chronic migraine.

**Table 1:** Summary of baseline patient demographics and characteristics

Baseline demographics and disease characteristics^**a**^	Episodic migraine *N* = 1338		Chronic migraine *N* = 836	
	Placebo (*n* = 894)	Galcanezumab 120 mg (*n* = 444)	Placebo (*n* = 558)	Galcanezumab 120 mg (*n* = 278)
Age (years), mean (SD)	41.9 (11.4)	40.9 (11.5)	41.6 (12.1)	39.7 (11.9)
Gender (female), n (%)	755 (84.5)	378 (85.1)	483 (86.6)	237 (85.3)
Race (white), n (%)	681 (76.2)	335 (75.5)	432 (77.4)	223 (80.2)
Ethnicity (not Hispanic or Latino), n (%)	677 (79.4)	342 (80.1)	401 (76.7)	195 (74.7)
Region, North America, n (%)	657 (73.5)	325 (73.2)	321 (57.5)	161 (57.9)
Duration of migraine disease (years), mean (SD)	20.5 (12.5)	20.5 (12.3)	21.9 (12.9)	20.4 (12.7)
Migraine headache days/month, mean (SD)	9.1 (3.0)	9.1 (3.0)	19.6 (4.6)	19.4 (4.3)
MIDAS total score, mean (SD)	33.1 (29.3)	31.9 (28.0)	68.7 (57.4)	62.5 (49.5)
MSQ total score, mean (SD)	58.8 (16.4)	58.2 (16.1)	44.4 (17.9)	45.2 (18.2)
Total monthly pain severity-weighted duration in hours, mean (SD)	123.9 (92.2)	122.4 (90.5)	321.2 (231.3)	324.5 (212.0)

Note: Daily severity-weighted duration was calculated as duration (in hours) x pain severity (none = 0, mild = 1, moderate = 2, severe = 3)

Abbreviations: *MIDAS* Migraine Disability Assessment, *MSQ* Migraine-Specific Quality of Life Questionnaire version 2.1, *SD* standard deviation

^a^Includes pooled data from two 6-month episodic migraine studies and one 3-month chronic migraine study

### Total pain burden

**Fig. 1:**
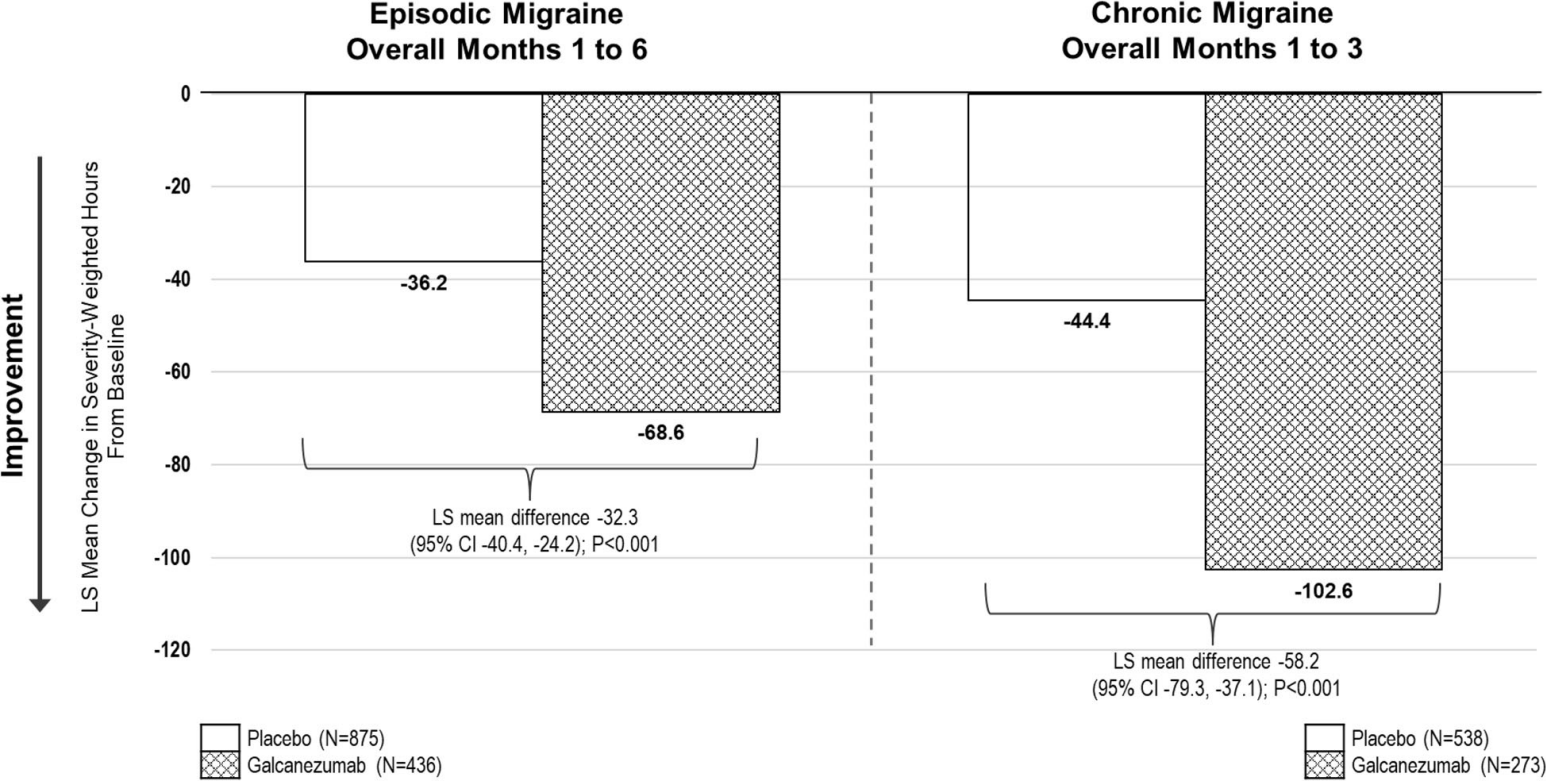
Total pain burden (monthly severity-weighted duration in hours) for patients with episodic migraine and chronic migraine. Greater decreases in total pain burden (severity-weighted hours) were observed with galcanezumab treatment Months 1 to 6 overall for patients with episodic migraine and Months 1 to 3 overall for patients with chronic migraine

The LS mean reduction from baseline in monthly total pain burden was greater in galcanezumab-treated patients compared with placebo-treated patients (Fig. 1). Starting at Month 1 in both the episodic and chronic migraine populations, the mean reductions from baseline were greater with galcanezumab compared with placebo and the same pattern was observed in all subsequent months of treatment (all *p* < 0.001) (data not shown). The overall monthly LS mean severity-weighted hours reduction from baseline across Months 1 to 6 for episodic migraine and across Months 1 to 3 for chronic migraine were greater for the galcanezumab groups compared with the placebo groups (*p* < 0.001; Fig. 1).

The average percent reduction from baseline in total pain burden in patients with episodic migraine across 6 months was greater with galcanezumab treatment (50.8%) compared with placebo (17.2%); mean difference was 33.6% (95% CI 41.3, 25.9; *p* < 0.001). Likewise, for patients with chronic migraine, the average percent reduction across 3 months was greater with galcanezumab (29.7%) compared with placebo (11.0%); mean difference was 18.7 (95% CI 25.6, 11.7; *p* < 0.001). As illustrated in Supplemental Material 1, for both the episodic and chronic migraine populations treated with galcanezumab, the percentage reduction in total pain burden was significantly greater than placebo starting at Month 1 (*p* < 0.001) and the significantly greater percentage reduction was maintained at each subsequent month (*p* < 0.001).

### Contribution of components to total pain burden

The duration of migraine on a given migraine headache day accounted for the greatest unique proportion of variability (episodic migraine studies, 57.4% and chronic migraine study, 61.1%) to the baseline total pain burden after adjusting for the frequency of migraine headache days and maximum pain severity (Table 2). In other words, the *duration* (hours) of migraine pain on the days when a patient had a migraine headache in a month contributed the most variability to total pain burden rather than the *number* of days with migraine headache in a month.

**Table 2:** Additional proportion of variability of the three components that contributed to total pain burden at baseline

Additional proportion of variability that contributed to total pain burden at baseline^a^	Episodic migraine^b^	Chronic migraine^c^
Frequency of monthly migraine headache days	22.6%	13.2%
Duration (hours per migraine headache days)	57.4%	61.1%
Severity of remaining migraine headache days	4.9%	4.8%

^a^Semi-partial provide the unique (or additional) proportion of variability explained by the particular variable in its ability to predict the outcome after accounting for the other 2 components

^b^Includes pooled data from two parallel 6-month studies

^c^Includes data from a 3-month study

Results from the sensitivity analyses related to pain severity scaling revealed that total pain burden remained significantly lower for the galcanezumab group compared with placebo even when using the square root of severity scores or the square of severity scores in the calculation of total pain burden. Thus, altering the severity rating scores on the calculation of total pain burden did not considerably alter the findings.

### Total pain burden correlations to MSQ and MIDAS

The MSQ and MIDAS scores at baseline were associated to total pain burden in the episodic (*r* = − 0.35 and *r* = 0.34, respectively; *p* < 0.0001) and chronic migraine (*r* = − 0.37 and *r* = 0.32, respectively; *p* < 0.0001) populations.

#### Change from baseline of individual components

The LS mean change for the individual components of total pain burden overall monthly across Months 1 to 6 for episodic migraine and Months 1 to 3 for chronic migraine are shown in Table 3. For both populations, the reductions from baseline in the frequency of, duration per, and the severity of the migraine headache days were significantly greater with galcanezumab treatment compared to placebo.

**Table 3:** Individual components of total pain burden - changes from baseline

	Episodic migraine Overall Monthly Across Months 1 to 6			Chronic migraine Overall Monthly Across Months 1 to 3		
Pain burden individual components, LS mean (SE)	Placebo (*n* = 872)	Galcanezumab (*n* = 435)	LS Mean Difference (95% CI)	Placebo (*n* = 535)	Galcanezumab (*n* = 273)	LS Mean Difference (95% CI)
Number of migraine headache days	−2.6 (0.2)	−4.5 (0.2)**	− 2.0 (− 2.4, − 1.6)**	− 2.7 (0.4)	−4.8 (0.4)**	− 2.1 (− 2.9, − 1.3)**
Hours per migraine headache day	0.1 (0.1)	−0.6 (0.1)**	− 0.7 (− 0.9, − 0.4)**	0.1 (0.1)	−0.5 (0.2)**	− 0.7 (− 1.0, − 0.3)**
Severity of remaining migraine headache days^a^	− 0.2 (0.02)	−0.2 (0.02)*	− 0.04 (− 0.1–0.0)*	− 0.1 (0.02)	−0.2 (0.02)**	− 0.1 (− 0.1, − 0.03)**

Abbreviations: *CI* confidence interval, *LS* least squares, *SE* standard error

*p < 0.05 versus placebo

**p ≤ 0.001 versus placebo

^a^Severity measured as: none = 0, mild = 1, moderate = 2, severe = 3

**Fig. 2:**
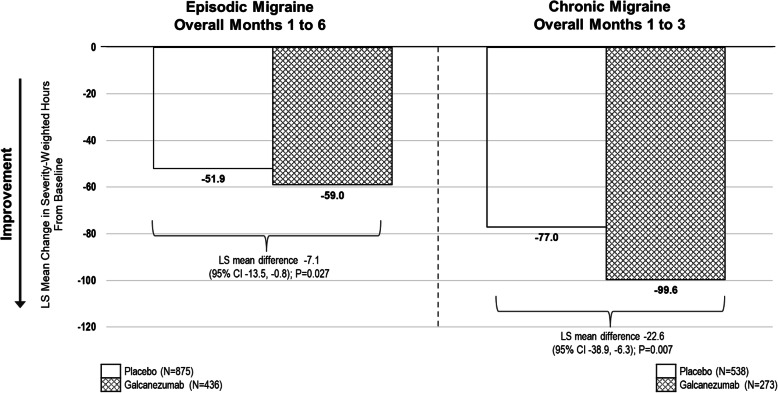
Total pain burden sensitivity analysis: total monthly severity-weighted duration in hours adjusted for change in migraine headache days for patients with episodic migraine and chronic migraine. Greater decreases in total pain burden (severity-weighted hours) were observed with galcanezumab treatment Months 1 to 6 overall for patients with episodic migraine and Months 1 to 3 overall for patients with chronic migraine

The sensitivity analysis conducted to explore the residual impact on total pain burden, even after controlling for the reduction of migraine headache day frequency, demonstrated that for both episodic and chronic migraine populations, the reduction in total pain burden was still greater in the galcanezumab groups compared with the placebo groups. In other words, galcanezumab treatment had impact on the other components of total pain burden (duration and severity) and supports findings from the analysis of the individual components of total pain burden (Table 3). Shown in Fig. 2 is the total pain burden (monthly severity-weighted duration in hours) adjusted for change in migraine headache days for patients with episodic and chronic migraine across months in the double-blind phase. The reduction in total pain burden was significantly greater in the galcanezumab groups compared with the placebo groups.

## Discussion

Treatment with galcanezumab 120 mg once-monthly injection (following a 240 mg initial loading dose) resulted in greater reduction of total pain burden relative to placebo. This study, combined with previous studies demonstrating the benefit of galcanezumab for reducing monthly migraine headache days, provides a more holistic view of the effect galcanezumab can have in patients with episodic or chronic migraine.

A composite measure of total pain burden produces a measure that may be more aligned to the personal pain experience in migraine. The almost exclusive focus on pain frequency as the metric for evaluating treatment “benefit” emanated from a desire articulated earlier this century for a standard, unified method for assessing treatment benefit [18]. In doing so, however, this may have inadvertently diminished the potential clinical value of assessing the impact of a treatment on the combination of frequency, severity, and duration of pain associated with migraine that may be more closely associated with the patient’s overall pain experience [19, 20]. Moreover, other “real-world” assessments of migraine include a variety of patient-reported outcomes (e.g., disability and quality of life) to fully assess the potential benefit of migraine treatment strategies [21, 22]. Total pain burden may better reflect what clinicians and patients discuss regarding the individual’s pain experience and could prove useful to further patient-centric discussions regarding treatment expectations when clinicians are evaluating options for migraine prevention. In this current analysis, all three components of total pain burden independently contributed to total pain burden; duration of migraine headache per day and number of monthly migraine headache days were the two most influential components. The current findings suggest that assessing total pain burden in a research or clinical setting allows for a more robust evaluation of the potential benefit of a preventive treatment for migraine (in this case, galcanezumab) in reducing the overall pain experience.

The evidence presented here suggests that galcanezumab could benefit a patient beyond reduction in migraine headache days per month. This observation may be especially relevant for those with chronic migraine as these patients will likely to continue to experience migraine headache more frequently than those with episodic migraine. If those remaining migraine headache days feature pain that lasts fewer hours and/or is less severe, then patients may be experiencing benefit that goes beyond those identified through unidimensional frequency counts.

The results support the hypothesis that total pain burden is a composite of various components that contribute unique information. Further, total pain burden is not simply a proxy to the number of migraine headache days since the reduction of total pain burden was greater with galcanezumab relative to placebo even after accounting for the reduction in monthly migraine headache days. This implies that the patient is experiencing an added benefit beyond experiencing fewer migraine headache days per month. Moreover, the positive correlation of total pain burden with MSQ and MIDAS further supports the construct validity of this composite measure and that disability is significantly correlated with total pain burden.

### Limitations

This study provides data regarding total pain burden in which published research pertaining to migraine is sparse. The outcomes from this study are limited by the post hoc nature of the evaluation and the clinical trial setting that restricts generalizability to the real-world population of patients with migraine. Another limitation of note may be the 4-point scale used to assess severity and its use in calculating the total pain burden given that a 4-point scale may have less sensitivity in detecting changes in severity than an 11-point scale. Finally, the scope of the analysis did not fully account for the influence of acute medication use on total pain burden.

The study outcomes are strengthened by the large sample size for both the populations of patients with episodic and chronic migraine and by the controlled nature of the study that allows for comparisons between groups providing greater confidence in considering the veracity of observed outcomes. Additionally, in the current analysis of total pain burden, we calculated severity-weighted hours such that 1 h of moderate pain was considered equally burdensome as 2 h of mild pain (similarly, this calculation considered 1 h of severe pain as equally burdensome as 3 h of mild pain). Others may consider different weighting algorithms to be more representative of total pain burden. However, our sensitivity analysis showed that changes in severity weights did not significantly alter the results when assessing the impact of galcanezumab on total pain burden.

## Conclusions

Greater reduction in total pain burden was seen in patients with episodic or chronic migraine treated with galcanezumab 120 mg once-monthly injection relative to placebo. This allows for a more robust evaluation of the potential benefit of preventive treatments (e.g., galcanezumab) in reducing the overall pain experience. Moreover, the discussion of total pain burden is well situated to support patient-centric discussions regarding treatment expectations when clinicians are evaluating options for migraine prevention.

## Supplementary information

Additional file 1. The video animations for the episodic migraine studies (Animation 1) and for the chronic migraine study (Animation 2) tracks the change in monthly total pain burden as a percent of baseline (y-axis) for each patient in the placebo (left, grey circles) and the galcanezumab 120 mg (right, red circles) group. The x-axis shows individual patients rank ordered by their final percent of baseline value at Month 6 for episodic migraine studies (Month 3 for chronic migraine study), ordered from greatest improvement on the left to greatest worsening on the right within each plot). Patients with missing data during the last month are placed towards the upper end of the x-axis. All patients start at a baseline of 100%. The solid purple line represents the mean percent of baseline in total pain burden within each treatment group at any point in time. In each scatterplot, black dotted reference lines are shown for baseline (100%) and 50% reduction from baseline in total pain burden. Any patient with a percent of baseline value below 100% has shown an improvement in total pain burden from baseline while patients with values below the 50% reference line have shown at least a 50% reduction in total pain burden compared to their baseline score. The bar plot in the middle shows the observed mean change in total pain burden from baseline to the end of each month for each treatment group (placebo, grey, left vs. galcanezumab 120 mg, red, right). The timeline bar across the top tracks the month of treatment being shown, from baseline (Month 0) to Month 6 for episodic (Month 3 for chronic) in the double-blind treatment phase.

## Data Availability

The datasets generated and/or analyzed during the current study are not publicly available due intellectual asset protection but are available from the corresponding author upon reasonable request.

## Abbreviations

CGRP: Calcitonin gene-related peptide
CI: Confidence intervals
EVOLVE-1, EVOLVE-2: 6-month, randomized, placebo-controlled studies (identical design) of galcanezumab in patients with episodic migraine
ITT: Intention-to-treat
LS: Least squares
MIDAS: Migraine Disability Assessment Scale
MMRM: Mixed-model repeated measures
MSQ: Migraine Specific Quality of Life Questionnaire
REGAIN: 3-month randomized, placebo-controlled study of galcanezumab in patients with chronic migraine
